# Ultrafast Dynamic Defect Inspection With Computational Neuromorphic Imaging

**DOI:** 10.1002/advs.202510338

**Published:** 2025-09-23

**Authors:** Shuo Zhu, Qianfeng Yin, Chutian Wang, Jianqing Huang, Edmund Y. Lam

**Affiliations:** ^1^ Department of Electrical and Electronic Engineering The University of Hong Kong Pokfulam Hong Kong SAR 999077 China; ^2^ Meta‐force Institute of Computation and Information (MICI) Hong Kong SAR 999077 China; ^3^ Hong Kong Industrial Artificial Intelligence and Robotics Centre (FLAIR) Hong Kong SAR 999077 China; ^4^ School of Aerospace Engineering Xiamen University Xiang'an South Road, Xiang'an District Xiamen Fujian 361005 China

**Keywords:** computational neuromorphic imaging, defect inspection, ultrafast dynamic environments

## Abstract

Inspecting surface defects is critical for ensuring quality in advanced manufacturing. However, current defect inspection techniques are largely limited to ideal imaging conditions and require vibration‐free environments when using traditional industrial cameras. These frame‐based cameras typically exhibit latency and a limited dynamic range, leading to vulnerabilities in production quality and restricting their efficiency in meeting increasing production demands. To address this challenge, a novel inspection paradigm is developed, using computational neuromorphic imaging (CNI) for ultrafast dynamic defect inspection. By leveraging the high temporal resolution and high dynamic range of event‐based sensors, CNI enables ultrafast, exceptional dynamic range yet cost‐effective defect inspection while minimizing perceptual and computational latency. Experimental results demonstrate event processing sampling times spanning 300‐fold in fast motion and dynamic ranges exceeding 10000‐fold under varying illumination. Additionally, event‐driven data facilitates direct edge detection and visualization of defects, which is critical for real‐time continuous diagnosis. Vibration is also utilized to enhance structural defect detection and improve robustness in practical settings. CNI offers unique advantages for industrial applications, enabling defect inspection across diverse products and complex conditions. This approach opens up a new route toward ultrafast industrial inspection in challenging environments, addressing the growing demands of mass real‐time inspection and intelligent diagnosis in precision manufacturing.

## Graphical abstract

Computational neuromorphic imaging (CNI) is leveraged for ultrafast dynamic defect inspection, offering unparalleled capabilities for fast‐moving samples, complex illumination conditions, and real‐time edge detection. The CNI paradigm will enhance our set of inspection tools in challenging environments, paving the way for intelligent, precise, and low‐latency industrial diagnostics and expanding potential imaging applications.

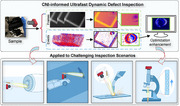

## 1 Introduction

The manufacturing industry is crucial, fueling innovations across various technology sectors and driving advancements in numerous fields. Consequently, industrial inspection is essential, involving meticulous examination of microchips to detect defects and ensure their quality, reliability, and performance.^[^ ^1^, ^2^, ^3^, ^4^, ^5^, ^6^ ^]^ For example, as the semiconductor industry advances along the trajectory projected by Moore's law and the growing market demand, conventional inspection solutions face mounting challenges in addressing manufacturing requirements.^[^ ^7^, ^8^ ^]^ Defect inspection in semiconductor manufacturing is an urgent step, identifying flaws such as scratches, pits, contaminants, and stains that compromise product integrity and functional performance. Advances in image sensors and integrated imaging techniques have enabled significant progress in addressing practical challenges across micro‐ to macro‐scale imaging applications.^[^ ^9^, ^10^, ^11^, ^12^, ^13^ ^]^ Consequently, industrial inspection leverages advanced technologies like optical microscopy, visualization systems, and intelligent algorithms to identify imperfections, playing an irreplaceable role in high‐performance manufacturing.^[^ ^14^, ^15^, ^16^, ^17^ ^]^ However, the process faces persistent challenges, particularly the urgent need to enhance efficiency, speed, and reliability simultaneously. Industrial products often incorporate materials with wide dynamic ranges, such as reflective metals and silicon, which can cause system uneven illumination. Additionally, the use of complex micro‐imaging systems and limitations posed by micro/nano‐scale prototypes may compromise signal capture efficiency. Concurrently, environmental factors like vibration, i.e., an unavoidable issue in micro/nano‐structures, further complicate the inspection process.^[^ ^18^ ^]^ Addressing these challenges necessitates the development of advanced defect inspection methodologies and intelligent diagnostic approaches.

Bio‐inspired neuromorphic sensors, also known as event cameras, are emerging imaging devices with more attractive properties than conventional sensors, which asynchronously capture per‐pixel luminance changes and offer attractive properties for advanced sensing and imaging super‐fast dynamics of motion targets.^[^ ^19^, ^20^, ^21^, ^22^ ^]^ Computational neuromorphic imaging (CNI), which integrates event cameras, optics, and computational models, represents a promising frontier in optical imaging. The CNI technique makes use of event sensors that encompass time‐efficient imaging, high dynamic range reconstruction, and high‐sensitivity sensing, and is ideally suitable for detecting ultrafast dynamic information and is robust to challenging environments. CNI encompasses ultrafast dynamic analysis, high‐sensitivity sensing, and energy efficiency, offering the transformative potential for academic research and industrial applications from micro to macro settings.^[^ ^23^, ^24^, ^25^, ^26^, ^27^ ^]^ The CNI‐informed approach merges neuromorphic vision sensors with advanced algorithms, overcoming the inherent limitations of traditional methods in capability and efficiency. The growing demand for energy‐efficient, high‐performance optical sensing, spanning machine vision to optical communication, necessitates innovation in spatiotemporal data processing at near single‐pixel scale.

For industrial inspection, conventional techniques encompass multi‐pixel photodetector arrays and frame‐based optical systems. State‐of‐the‐art frame‐based inspection methods, even those using advanced learning techniques, are data‐driven and rely on high‐quality images. Their effectiveness hinges on the availability of such images, limiting performance when image quality is compromised.^[^ ^28^ ^]^ These methods often struggle to achieve rapid, real‐time detection and processing of dynamic targets within the sensor. Challenges escalate in handling high‐dimensional spatiotemporal data, where dependence on sequential data input and external processing introduces latency, reduced throughput, and higher energy consumption, hindering parallel real‐time capabilities.^[^ ^26^, ^29^, ^30^, ^31^ ^]^ The CNI‐informed system achieves initial sample focusing in milliseconds,^[^ ^32^, ^33^ ^]^ enhancing efficiency across manufacturing and diagnostic workflows. Event cameras also detect subtle surface light intensity changes caused by equipment vibrations, visually representing mechanical behaviors.^[^ ^34^, ^35^ ^]^ Consequently, the Bio‐inspired system possesses an efficient information perception and computational capability, which is crucial in addressing the aforementioned challenges in imaging and vision. While event cameras have been widely used in computer vision and robotics, their application to micron‐level industrial defect inspection is a new frontier with unique challenges. Our CNI approach addresses these challenges by integrating event cameras with a high‐magnification optical system and advanced computational models. Unlike conventional frame‐based cameras, which struggle with motion blur and limited dynamic range in such conditions, our method excels in these demanding scenarios.

To achieve this, we develop an ultrafast dynamic defect inspection method that achieves exceptional temporal resolution, enabling robust detection under varying illumination. Combining an event camera with an optical microscopy system, the CNI‐informed method leverages high temporal and high dynamic resolution to enhance inspection capability and efficiency while minimizing latency. Additionally, event characteristics can be used for direct defect visualization along the edges, a critical link in real‐time continuous defect diagnosis. Furthermore, vibration data analysis extracts structural defect features, improving detection robustness. This approach uncovers previously undetectable defects, surpassing conventional characterization limits. With its high speed, low latency, and wide dynamic range, the CNI‐informed method offers unique advantages for imaging faint defects and applies to diverse industrial products and complex scenarios.

## 2 Results

### 2.1 Design of the CNI‐Informed Inspection System

Event cameras represent a transformative imaging paradigm, capturing dynamic signals with low power consumption and versatile processing capabilities.^[^ ^36^ ^]^ This paradigm enables ultrafast dynamic responses, overcoming inherent limitations in signal accuracy and misjudgment faced by conventional methods. Defect signals are initially converted by bio‐inspired circuits and then processed in real‐time with inspection assistance. Our reported approach demonstrates the CNI‐informed defect inspection of sophisticated structures within different samples, under complex environments, enabled by cost‐efficient yet powerful event cameras. Subsequently, leveraging these attractive properties of neuromorphic sensing, the CNI framework surpasses traditional frame‐based systems in accuracy and efficiency, particularly in challenging inspection environments.

**Figure 1:**
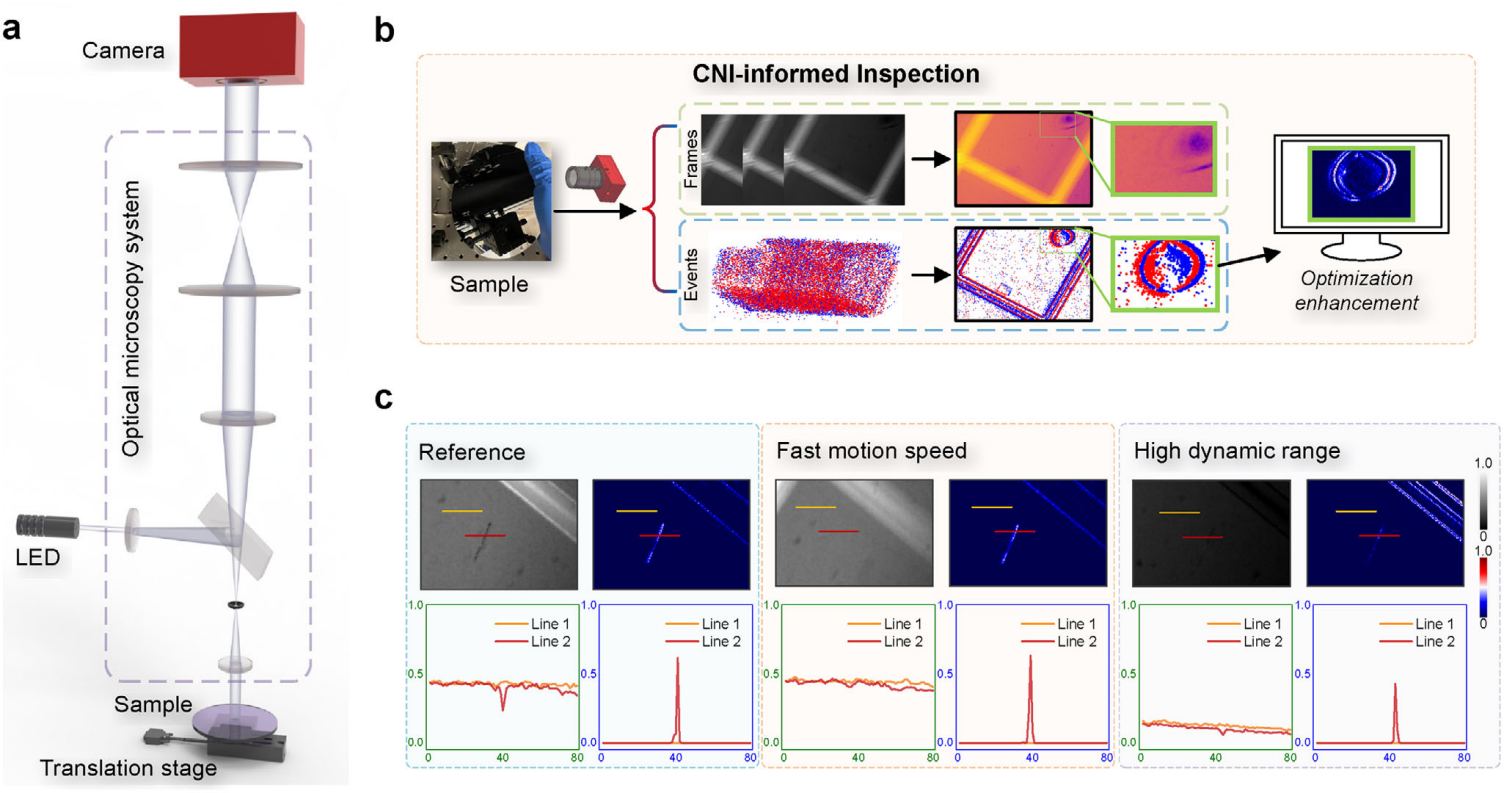
Schematic of ultrafast sensing and inspecting defects using an event camera. a) The optical setup used for defect inspection. A camera with simultaneous events and frames output is embedded into a bright field microscopy system for moving sample inspection. b) The output frames and event data, and the inspection workflow for the fast‐moving sample. The presented defect is a stain on the chip. c) The defect visualization with collected frames, accumulated events, and the corresponding normalized intensities of the line in different regions. The presented defect is a small scratch on the chip, and our method has better and more robust visibility.

As illustrated in **Figure** **1**a, we design the CNI‐informed dynamic inspection system integrating an event camera with an optical bright‐field microscopy system, employing a coaxial forward illumination scheme. More experimental hardware information can be found in Methods and Note S1 (Supporting Information). Experimental data of semiconductor chip inspection are presented in Figure 1b for the defects visualization, which is collected by the developed CNI‐informed optical system. Inspired by the neural architecture that might enable more efficient analysis and computation with differential approaches, our inspection system is sensitive to dynamic signals. Compared to discrete conventional frames, we can obtain a continuous event stream within the CNI framework. An event *e*_*k*_ = (**x**_*k*_, *p*_*k*_, *t*_*k*_) is triggered when the logarithmic brightness change *L*(**x**_*k*_, *t*_*k*_) exceeds a preset threshold *C* at the timestamp *t*_*k*_

(1) $$\Delta L\left(x_{k},t_{k}\right)≜L\left(x_{k},t_{k}\right)-L\left(x_{k},t_{k}-\Delta t_{k}\right)=p_{k}C$$

where **x**_*k*_ = (*x*_*k*_, *y*_*k*_) is the spatial coordinates, and Δ*L*(**x**_*k*_, *t*_*k*_) represents the recorded temporal increment with the time elapsed Δ*t*_*k*_ since the last event at the same pixel **x**_*k*_.^[^ ^37^, ^38^ ^]^ During the inspection process on the production line, defects typically manifest alongside the generation of abnormal events. Therefore, event‐based defect detection exhibits superior sensitivity and robustness compared to traditional frame‐based methods, particularly in situations involving high‐speed motion or high dynamic range challenges. Benefiting from the advanced properties and unique response mechanism of event cameras, defects can be identified even in complex inspection conditions, such as those involving fast‐moving samples or requiring high dynamic range sensing.

### 2.2 Dynamic Defect Visualization With the CNI Framework

Timely detection and visualization of semiconductor defects are critical in product manufacturing and diagnostics, particularly for subtle flaws such as light scratches, micro‐pits, and low‐contrast contaminants, which are challenging to identify under fast dynamics or complex inspection conditions. Addressing these challenges requires developing advanced detection schemes to ensure reliability. Manufacturers prioritize robust defect detection to mitigate economic losses and reputational risks. As shown in Figure 1b,c, the CNI‐informed method excels in diverse and challenging defect inspection scenarios. In situations involving fast motion and high‐dynamic manufacturing environments (such as low light or excessively bright lighting conditions), it is challenging to visualize and extract blemish features, making them relatively difficult to detect and diagnose. Despite the improved defect contrast offered by the coaxial forward lighting scheme,^[^ ^39^ ^]^ the event camera, with its superior responsiveness to moving targets and logarithmic response mechanism, broadens the dynamic range of target detection. This makes defect inspection more efficient and precise, particularly in extreme manufacturing environments.

In contrast to standard production areas, defect generation results in unique and significantly different events within the detection framework of the CNI paradigm. This can aid us in rapidly, efficiently, and accurately inspecting defect‐related information. As illustrated in Figure 1b, the overall structural details of the wafer or chip are predefined, allowing predictable event patterns during motion diagnostics. This predictability enables precise spatial localization and parameter extraction from defect‐related events, distinguishing them from normal product signals. As illustrated in Figure 1c, our method for defect sensing and inspection exhibits enhanced responsiveness, with characteristics that are more distinct than those observed in conventional approaches. The drawn lines depict the normalized intensities of the image, and the curves in the figure show that our method enables a clearer and more explicit characterization of the defects, making them easier to detect. These results indicate that the CNI‐informed scheme facilitates easy differentiation between defects and backgrounds. Successful defect inspection is also confirmed by Video S1 (Supporting Information), which visualizes the defects of fast‐moving samples generated from each output event according to the abnormal dynamics. Therefore, we can harness the advanced dynamic response properties of event cameras for challenging defect inspection, validating it across various demanding manufacturing environments.

### 2.3 Experimental Validation of CNI‐Based Defect Inspection

We have showcased the CNI‐informed inspection method across various challenging environments, including high‐speed detection, high dynamic range sensing, direct edge visualization, and using vibration for defect inspection. Preliminary experimental results indicate that the CNI‐informed approach can be harnessed as a straightforward yet potent technique for ultrafast dynamic defect inspection. It transcends traditional limitations and transforms drawbacks into advantages.

**Figure 2:**
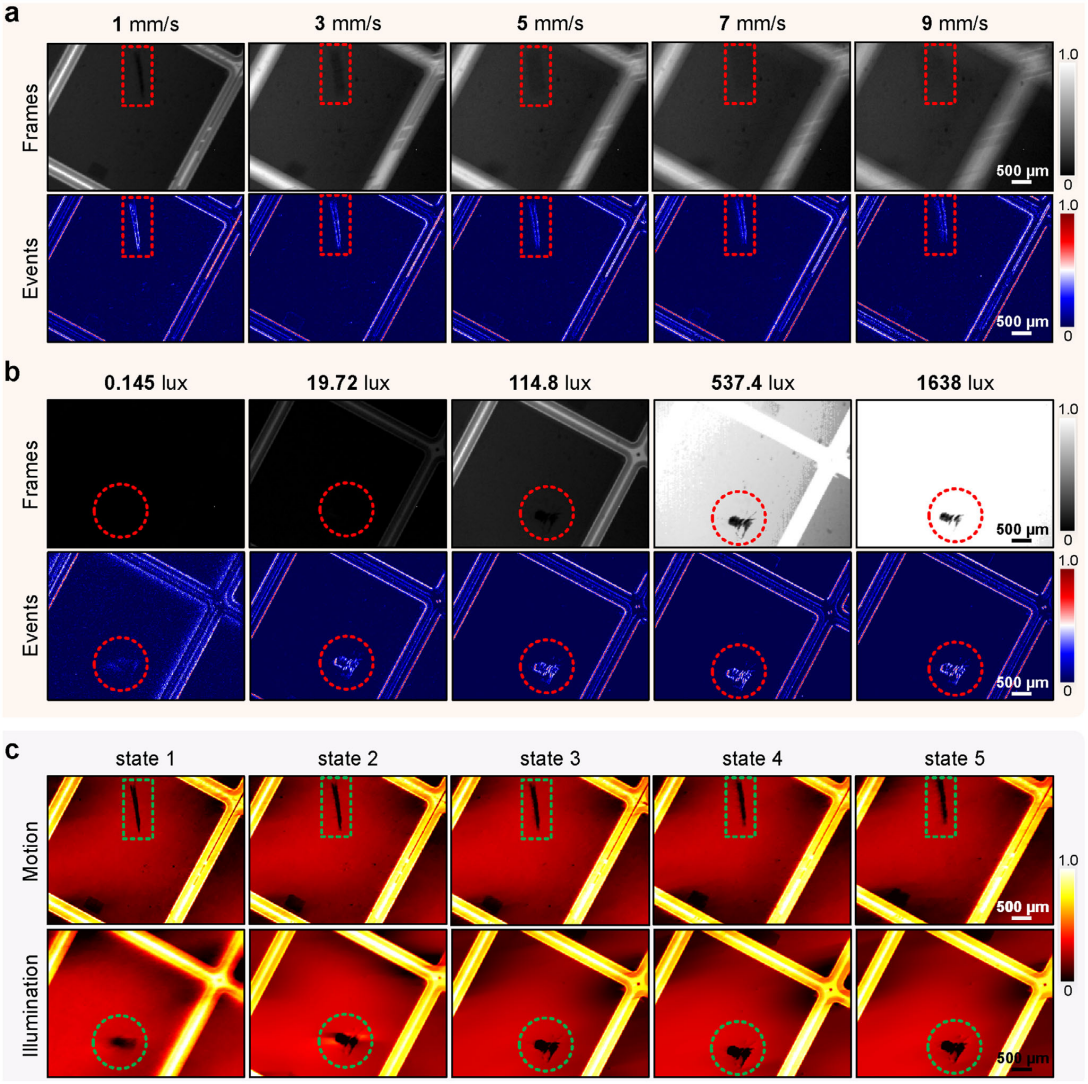
Defect inspection for dynamic samples using event sensors. a) and b) are direct data visualizations with frames and events. a) Data visualization results of dynamic defect at different motion speeds (i.e., 1, 3, 5, 7, and 9 mm/s, respectively) in frame and event modes. From left to right, the speed of movement gradually increases. b) Data visualization results of dynamic defects at different illumination conditions with frame and event modes. From left to right, illumination conditions from low light to over‐bright lighting (i.e., 0.145, 19.72, 114.8, 537.4, and 1638 lux, respectively). c) Defect image reconstruction using pure events with our method. Scale bars: 500μm.

High‐speed motion detection of semiconductor chips is critical for enhancing production efficiency. With their superior motion perception, event cameras outperform traditional cameras in diagnosing defects in moving samples. **Figure** **2**a displays the data‐only connection visualization results for different moving samples. These results reveal that, at low speeds, traditional frame‐mode cameras can effectively visualize defects and extract defect features. The exposure time of frame mode is set as 0.1 s (i.e., 10 fps), and the initial time windows of the accumulated event are also set as 0.1 s. However, as sample speed increases, traditional detection methods struggle to adapt to high‐speed motion diagnosis scenarios. In contrast, event cameras continue to deliver reliable diagnostic results.

The CNI‐based approach significantly improves the efficiency and accuracy of defect inspection for fast‐moving semiconductor chips while ensuring robust environmental resilience. The reflection coefficients of semiconductor chips made from various materials can vary widely. Integrated circuit boards of different types usually exhibit varying reflection characteristics. For instance, semiconductor chips, which possess high reflection coefficients and ultra‐smooth surfaces, are susceptible to over‐exposure or under‐exposure when the installation is uneven or the packaging is improperly adjusted. These issues are exacerbated during high‐speed motion detection, where motion device stability and flatness can further challenge the effective dynamic range of detection. While current deblurring methods offer limited improvements for fast‐motion samples,^[^ ^40^ ^]^ their effectiveness declines as motion speed increases. Additional comparative experimental results are provided in Note S8 (Supporting Information).

Event cameras offer a very high dynamic range (i.e., up to 140 dB than conventional 60 dB), which extends the sensing capability in extremely dark and bright environments with CNI‐informed approaches.^[^ ^41^, ^42^ ^]^ As illustrated in Figure 2b, the event camera can effectively visualize sample defects under varying exposure conditions and extract defect features in different extreme illumination environments. In contrast, traditional frame cameras often fail to diagnose defects effectively, leading to significant information loss in both dark and bright scenes. The results presented in this paper confirm that, owing to their logarithmic response characteristics, event cameras exhibit heightened sensitivity to visualize defects in extremely bright and low‐light environments. The CNI‐based defect inspection method demonstrates superior sensitivity and diagnostic capabilities in low‐light conditions, aligning well with the current emphasis on low power consumption and high efficiency in production settings. Our inspection method with event processing in the dynamic range can span 10000‐fold in different illumination conditions. Therefore, our technique is feasible to maintain inspection robustness for reflective properties of different sample materials, making it ideal for complex, multi‐category defect inspections. Concurrently, the concept of the dark factory has gained popularity, emphasizing full automation and requirements for low‐power consumption as well as other aspects of computation and energy efficiency. For final image reconstruction, we use pure events to enhance the visual performance of defect images. As shown in Figure 2c, our method enables the reconstruction of images at high motion speeds and with high dynamic ranges in illumination environments (i.e., extremely dark and bright settings). The detailed introduction of reconstruction algorithms is presented in the Methods part and Notes S2 and S3 (Supporting Information). These results facilitate precise sample analysis and diagnosis, underscoring the method's potential for advanced industrial applications. Additional results under challenging illumination conditions are presented in Note S4 (Supporting Information). The CNI‐informed approach can handle varying sample sizes, surface roughness, and reflectivities with great imaging capability, which maintains performance across surface finishes. More examples are presented in Note S9 (Supporting Information).

**Figure 3:**
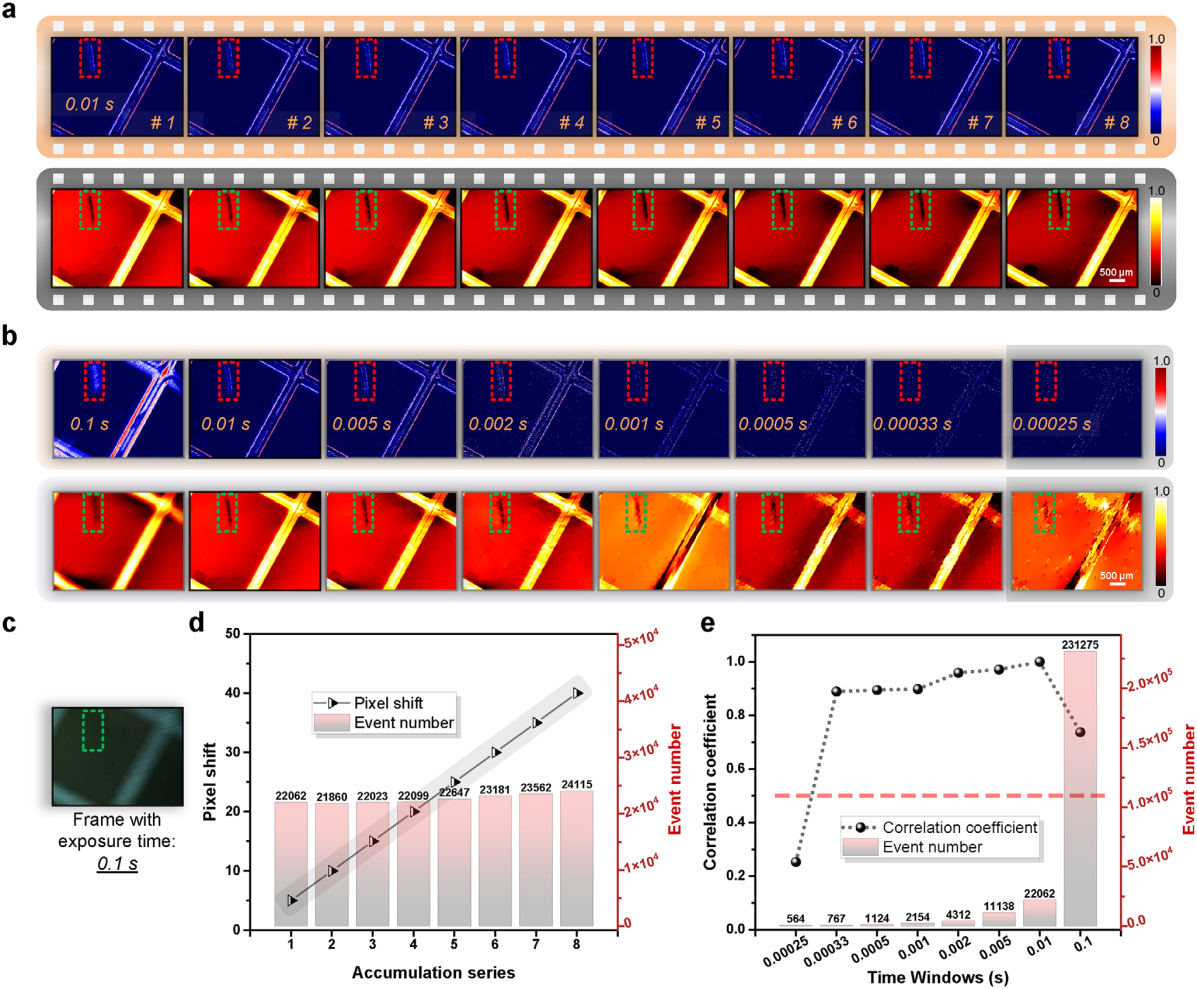
Analysis for ultrafast defect inspection with subdivision of time window. a) Events and reconstruction results for the continuous time windows division. The presented events and figures are subdivided from the duration of the corresponding frames with a fixed exposure time. b) The defect data visualizations with warped events and image reconstructions with pure events. Events visualization and reconstruction results of dynamic defect at motion speed 10 mm/s, are with the predefined time window of 0.1 s. Hence, the time windows of the presented results are 0.1 s, 0.01 s, 0.005 s, 0.002 s, 0.001 s, 0.0005 s, 0.00033 s, and 0.00025 s, respectively. c) The blurry image with conventional frame mode and the exposure time is 0.1 s. d) The evolution of the pixel shift with the first starting position and corresponding event number. e) The evolution of the correlation coefficient with the second predefined image and the corresponding number of used events. Scale bars: 500μm.

We further analyze the ultrafast temporal resolution with our method in **Figure** **3**, which contains detailed results about the performance with the time window and event number. Here, the CNI‐informed approach captures a continuous stream of high‐temporal resolution events, equivalent to a single conventional frame that captures a blurred defect image. These events are decomposed into discrete temporal windows for subsequent analysis and reconstruction. As shown in Figure 3a, decomposing the event stream data, equivalent to the data from multiple conventional frames, yields a sequence of high‐resolution defect images. This method also facilitates dynamic tracking of defect positions across temporal windows. The displacement results in Figure 3d closely match the linear motion of the system's platform, validating the method's accuracy when incorporating the optical system's magnification and motion stage parameters. The exposure time in traditional camera mode remains consistent, i.e., 0.1 s. As the object's motion speed increases to 10 mm/s, images captured by traditional methods exhibit severe blurring, rendering them ineffective for defect analysis. In contrast, the collected event data forms a continuous, finely divisible stream with high temporal resolution. By selecting varying time windows, we can visualize and reconstruct defects with high precision. As shown in Figure 3b, the initial time window is set to a predefined accumulation time with 0.1 s divided by 10 (i.e., 0.01 s), with subsequent windows progressively reduced by factors of 1, 20, 50, 100, 200, 300, and 400 (i.e., 0.1 s, 0.005 s, 0.002 s, 0.001 s, 0.0005 s, 0.00033 s, 0.00025 s). Even with significantly fewer events (i.e., down to 0.33% of the original data), the approximate shape and relative position of defects remain discernible. Correlation coefficients are calculated for defect images reconstructed under ideal conditions using varying fractions of the total event data. As shown in Figure 3e, when reconstructing with only 1/400 of the events, the correlation coefficient falls below 0.5, indicating unreliable defect detection. However, increasing the event count to 1/300 achieves a correlation coefficient of 0.9, enabling robust defect reconstruction. The proposed method demonstrates a 300‐fold improvement in temporal coverage compared to prior approaches, significantly enhancing the reliability and accuracy of defect analysis over extended time spans. The event processing speed of this experiment can span 300‐fold for ultrafast moving samples. Integrating artificial intelligence techniques could enhance the method's performance in such scenarios.^[^ ^43^, ^44^ ^]^ Therefore, this approach demonstrates a substantial improvement in defect diagnosis with ultrafast speed and flexible performance compared to traditional methods.

**Figure 4:**
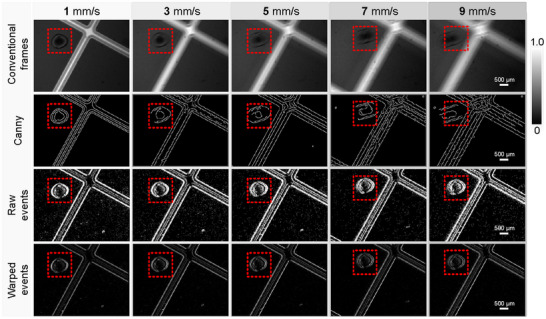
Directly edge visualization of defects using CNI‐informed inspection. The first row displays the samples to be examined at varying motion speeds, the subsequent three rows illustrate the edge information obtained through the utilization of Canny's algorithm with frames, and the third and fourth rows present the outcomes of the samples represented directly through raw events and warped events. Scale bars: 500μm.

Edge detection is a fundamental challenge in computer vision and image processing, critical for simplifying image analysis, enhancing interpretability, and enabling downstream applications.^[^ ^45^ ^]^ In industrial inspection, edges serve as key indicators for defect identification, segmentation, and analysis. Rapid and accurate edge detection is a practical tool to address numerous inspection problems, while neuromorphic computing can provide a powerful way to precisely solve this point.^[^ ^19^, ^46^, ^47^, ^48^ ^]^ Events aligned with motion inherently capture sharp, edge‐coincident spatial structures.^[^ ^49^ ^]^ The event camera, unlike the traditional frame‐based camera, generates sparse event data at a pixel whose logarithmic intensity changes, and the corresponding event is generated in a nearby pixel with the shape of an edge that can be preserved.^[^ ^50^ ^]^ Here, we employ contrast maximization (CM) as an optimization framework to refine these structures, aligning spatiotemporal event data with moving intensity gradients.^[^ ^51^, ^52^ ^]^ As shown in **Figure** **4**, the edge of the defect can be directly visualization using the raw events and enhanced edges with the image of warped events within the CNI paradigm. The edge information of ultrafast dynamic defects can be displayed for real‐time diagnosis with millisecond‐aligned events. The developed technique utilizes the captured events directly to characterize yet appear to be edge information and to visualize defect edges and features concisely and efficiently with a simple warp. The experimental results demonstrated that our edge visualization and detection method is robust under various moving conditions. The experimental results demonstrate the efficacy of our developed method in detecting direct edges in complex dynamic scenes, with minimal motion blur in the obtained edge information. Note S5 (Supporting Information) provides additional edge visualization results and analyses on different samples, further validating the efficacy, robustness, and expandability of our method.

**Figure 5:**
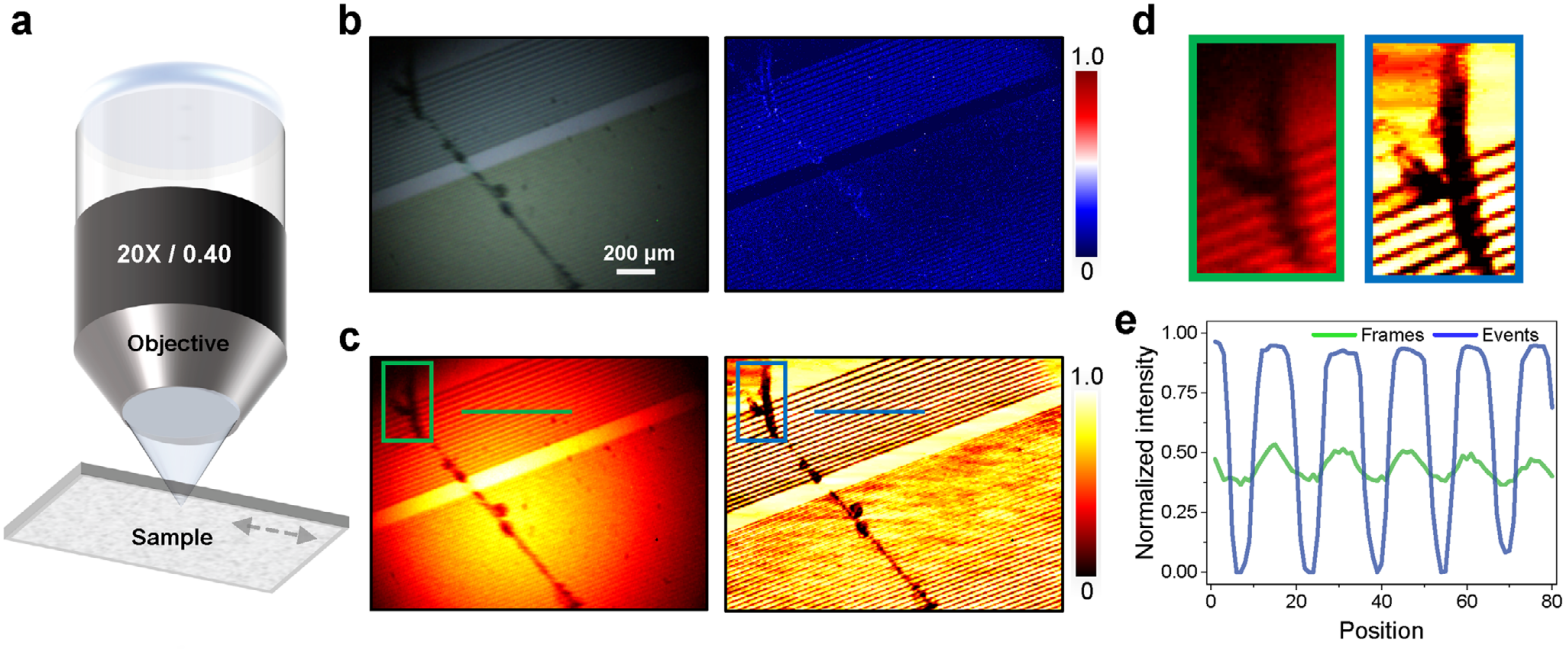
Harnessing vibration for defect inspection under the higher magnification. a) An alternative 20X objective is used to verify the inspection performance under vibration in a larger magnification. b) The raw collected frame data and events with vibration. c) The normalized frame and reconstruction with pure events. d) Zoom‐in images of the defective area of interest in subFigure c). e) Normalized intensity at the descriptive lines in subFigure c). Scale bars: 200μm.

In traditional optical inspection, vibration is typically considered a detrimental factor, compromising the accuracy and reliability of detection and diagnosis.^[^ ^53^ ^]^ This is particularly problematic in high‐resolution optical systems, where even minor vibrations can cause motion blur and hinder quantitative measurements. Here, we introduce the use of vibration with the CNI approach to combat blur in practical scenes. In this context, the relative motion induced by vibration can be utilized to enhance image resolution by using the correlated events containing sample structure information for high‐magnification defect diagnosis. To reconstruct defect images using vibration, we employ the same method as the above that utilizes micro‐motion triggered events. Benefiting from the high temporal resolution events, we precisely use events generated by unidirectional movement displacements within one cycle for IWE and image reconstruction. As demonstrated in **Figure** **5**, the introduction of external vibration causes the frame‐mode camera of the transmission to produce motion blur, thus reducing the contrast of the captured image and affecting the precise detection of the sample and the visualization of defects.^[^ ^54^ ^]^ We employ the events generated by the vibration to facilitate event characterization and reconstruct structural information pertinent to sample defects. The reconstruction results show that the structural information of the chip can be efficiently reconstructed, thanks to the high temporal resolution capability of the time camera. From Figure 5c,d, it is evident that image reconstruction and defect detection based on vibration events provide better visualization and resolution compared to traditional frames. Additionally, the corresponding visualization results indicate that the event‐based method offers superior resolution capability compared to frame‐mode data. More vibration results and analysis are provided in Note S6 (Supporting Information).

## 3 Discussion

We have showcased our ultrafast dynamic inspection technique that employs CNI technique to eliminate dynamic‐induced artifacts, eliminating dynamic‐induced artifacts across various distinct environments. However, the CNI‐informed framework is versatile and should apply to a variety of industrial inspection methods. By capitalizing on the high temporal resolution and high dynamic range of events, this approach provides distinct advantages in imaging subtle defects, thus enhancing defect detection across a broader range of industrial products and complex inspection scenarios.

**Figure 6:**
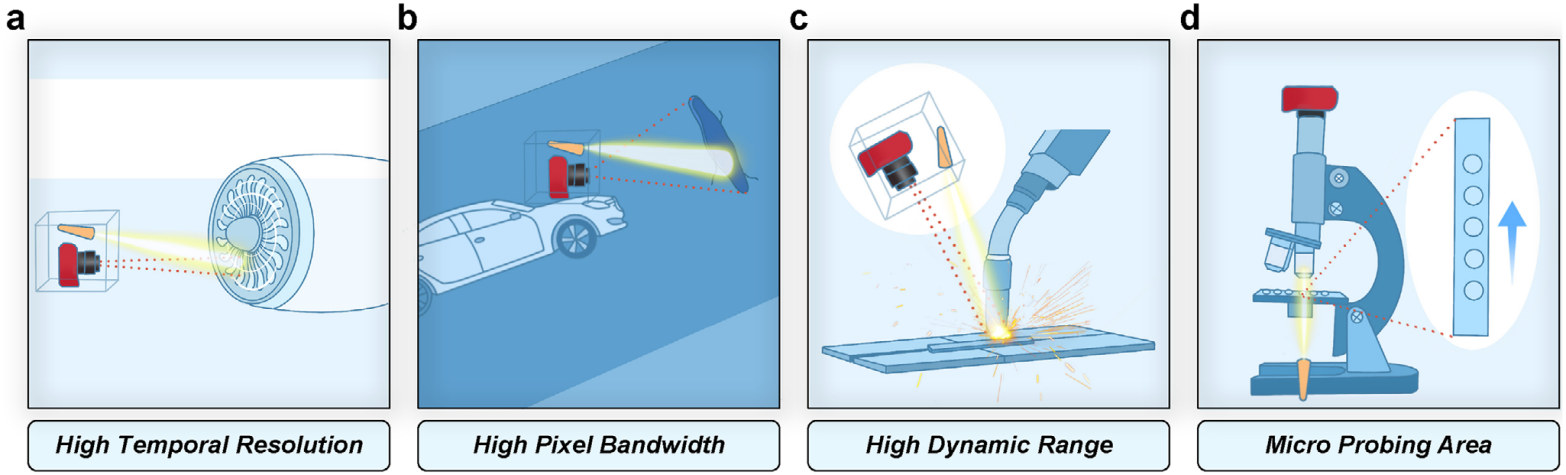
Key attributes and potential future applications of CNI‐informed inspection. The CNI approach combines attractive attributes highlighted in the following potential future defect inspection applications: in each example, event cameras are used to capture ultrafast dynamics under challenging scenarios. a) The high temporal resolution allows for defect inspection in high‐rotated motion (e.g., running aircraft turbine inspection). b) The high pixel bandwidth allows real‐time defect data transmission (e.g., road surface inspection). c) The high dynamic range allows challenging manufacturing monitoring (e.g., weld metal inspection). d) The small probing area allows mesoscopic/microscopic dynamics detection (e.g., biomedical tissue inspection).

While CNI‐informed inspection is a powerful tool for analyzing ultrafast dynamic scenes using continuous event data, its effectiveness depends on motion and variation, with performance governed by temporal resolution and logarithmic response. In contrast, frame‐based sensors frequently grapple with latency and a constrained dynamic range, rendering them ill‐suited for extreme inspection scenarios. Additionally, traditional methods face trade‐offs in the field of view, resolution, and detection efficiency. As illustrated in **Figure** **6**, the CNI‐informed inspection holds wide‐ranging potential applications. For example, this method can be used to inspect running aircraft turbines with high temporal resolution, which is crucial for defect inspection in high‐rotation motion. As it is feasible to allow the system to dynamically autofocus and capture circular motion‐induced events that the image plane is accurately obtained and motion is effectively frozen, ensuring that defects are visible without blurry artifacts. Second, high pixel bandwidth is essential for real‐time defect data transmission, enabling the system to quickly send and process large amounts of image data. This is particularly important in applications where immediate action is required to address detected defects, such as round‐the‐clock road surface inspection with a high‐speed working vehicle. Third, benefiting from the robustness capability to high dynamic scenes, our technique can handle defect inspection under varying light conditions, making it useful in manufacturing environments like weld metal inspection. Fourth, it can focus on small details with high sensitivity, making it ideal for biomedical tissue inspection at mesoscopic and microscopic levels. Despite its advantages, challenges remain, and further optimization of event‐based techniques is needed for specialized applications.

Within the CNI paradigm, there is scope to explore more implementations of advanced imaging and optical modulation among challenging academic research and numerous industrial applications, ranging from micro‐ to macro‐level scenarios (e.g., compressed ultrafast photography, microfluidics, and dynamic biomedical imaging).^[^ ^27^, ^55^, ^56^, ^57^ ^]^ In addition to optical modulation and systems to refine the CNI paradigm in such micron‐level scenarios. Integrating advanced intelligence algorithms is a powerful way to further improve CNI techniques.^[^ ^58^, ^59^ ^]^ For example, to design more efficient learning‐assisted CNI techniques, spiking neural networks (SNN) are well‐suited for processing event‐based data due to their ability to handle asynchronous, sparse inputs efficiently. By leveraging SNNs, we can potentially improve robustness with sparse event data by learning temporal correlations and spatial relationships. Future work will explore optical modulation and SNN integration into our CNI framework, which could further enhance robustness with event stream data.

## 4 Conclusion

In summary, we have developed an ultrafast dynamic inspection technique for defect feature visualization, validated through proof‐of‐principle experiments. The unique inspection philosophy inherent in the CNI paradigm renders it ideal for challenging dynamic environments where conventional methods fall short. Using events facilitates direct edge visualization of defects, which is instrumental for real‐time and robust defect inspection. Additionally, we harness vibrations to derive informative aspects for the event generation of structural defects. The CNI‐informed method reveals previously undetectable features, enabling unprecedented sample characterization. These capabilities are essential for downstream tasks and advanced inspection analysis. Our work establishes a new paradigm for industrial inspection, addressing the growing demand for real‐time mass quality control and intelligent diagnostics in manufacturing.

## 5 Experimental Section

### Experimental Setup

This study applies the CNI method and computational algorithm for ultrafast defect inspection using an event camera with an optical microscopy system. The designed setup is shown in Figure 1a, which combines an event camera and a bright field microscopy system for semiconductor defect inspection. A simple commercial bright field microscope system is employed as the optical system, and an event camera is selected as the signal detector. We use an event camera (iniVation, DAVIS 346, 346 × 260 pixels) with a time resolution of approximately 1μs per single event, and the dynamic range of event mode is about 120 dB. DAVIS 346 also supports simultaneous frame and event output. The measurement rate of frame mode is up to 40 fps, and the corresponding dynamic range is about 40 dB. A white LED is employed as the light source to illuminate the defect sample, which can adjust the luminance for different illumination conditions. A semiconductor chip is selected as the inspection sample and is mounted on the linear translation stage (WN262TA20, Winner Optics). Here, we employ the translation stage for a single‐direction motion and dynamic defect inspection. More detailed system descriptions, discussion of hardware, and potential expansion with different setups are provided in Supplementary Note 1.

### Data Acquisition

The samples were tested at varying speeds of 1, 3, 5, 7, and 9 mm/s respectively. The first type of motion, i.e., the speed with 1 mm/s, is with the time window for accumulating events at 0.1 s, and the exposure time of frames is the same as it. Beneficial from the high temporal resolution of events, the time windows of other motion states are proportionately reduced with the speed increase, and the rates are inversely proportional to the magnitude of the speed. The variable time windows so that the number of events used is essentially the same. For varying cases of illumination brightness, we also employed five different intensity modes and maintained the movement speed at 2 mm/s during data acquisition. During the high dynamic range tests, we used a photometer (TES‐1339R, measuring levels ranging 0.01 to 999.900 lux, resolution: 0.01 lux) to quantitatively calibrate the illumination conditions of the inspection environment. We opted to generate events using the vibration produced when the motor is immobilized in a fixed position. An alternative 20X objective is employed to further verify the inspection performance, introducing more vibrations to the subsequent effect with a larger magnification. Different motor speeds correspond to different vibrations, yet no stage displacement, and in this case, we selected the vibration frequency generated at a speed of 0.5 mm/s for event generation and data acquisition. The RGB images in this work are converted into grayscale images for corresponding post‐processing and comparison.

### Data Processing and Defect Reconstruction

In the optimization enhancement process of practical event stream data, the time duration Δ*t* is set as $1\times 10^{5}\;\mu s$. The frame exposure time is the same as the event accumulation time and the frame interval is set to be zero, for a fair comparison with the event‐based method. Here, we first tackle the combined problem of motion and brightness estimation, which leads us to formulate event‐based image reconstruction as a linear inverse problem.

The estimated optimal flow can be solved by maximizing the sharpness of the image of warped events (IWE), which is characterized by the variance

(2) $$v^{*}=\max_{v}\frac{1}{\left|\Omega \right|}\int_{\Omega }{\left(\Delta \hat{L}\left(x;v\right)-\mu \left(\Delta \hat{L}\left(x;v\right)\right)\right)}^{2}dx$$

where |Ω| is the area within the sensor boundary, $\Delta \hat{L}$ is the IWE by accumulating events along the point trajectories, and μ(·) represents the statistical mean.^[^ ^51^ ^]^ Subsequently, as the clearest IWE provides an estimation of the motion‐corrected increment within Δ*t*, its spatial distribution can be physically interpreted as the gradient according to the known optical‐flow constraint^[^ ^37^ ^]^

(3) $$\Delta \hat{L}\left(x;v^{*}\right)\approx \Delta L\left(x\right)=-\nabla L\left(x\right)\cdot v^{*}\Delta t$$

As such, the optimal logarithmic intensity of the defect image can be solved by minimizing the data fidelity cost function

(4) $$\begin{array}{ccc} L^{*}\left(x\right) & = & \text{arg}\min_{L}\frac{1}{2}\left|\right|\Delta \hat{L}\left(x;v^{*}\right)\\ & & +\nabla L\left(x\right)\cdot v^{*}{\Delta t||}_{2}^{2}+\kappa_{1}{\left||\nabla L\left(x\right)|\right|}_{1}\\ & & +\kappa_{2}\left|\right|\nabla^{2}L\left(x\right){\left|\right|}_{1} \end{array}$$

where || · ||_*p*_ represents the *p*‐norm, and κ_1_, κ_2_ are the weights of the regularizers. Here, the selected hyperparameters of the regularizer weights are κ_1_ = 0.05, κ_2_ = 0.01, respectively. More details about the effect of optimized parameters are provided in Note S3 (Supporting Information). We can obtain the optimized signals for defect inspection and further analysis, and the IWE has sharp visualization to represent the sample edges well, which is helpful for real‐time diagnosis.

## Conflict of Interest

S.Z., Q.Y., C.W. and E.L. are co‐inventors of a pending patent application on the presented method. The remaining authors declare no competing interests.

## Author Contributions

S.Z. initiated this line of investigation, designed and implemented the project. S.Z., Q.Y., C.W. and E.L. contributed, developed, refined the concept and wrote the paper. E.L. supervised the project and funding acquisition. All authors reviewed the manuscript.

## Supporting information

Supporting Information

Supplemental Video 1

## Data Availability

The data that support the findings of this study are available from the corresponding author upon reasonable request.
