# Supplementary material for: Ultrafast Dynamic Defect Inspection With Computational Neuromorphic Imaging
Source: Adv Sci (Weinh). 2025 Sep 23;12(44):e10338. doi: 10.1002/advs.202510338 (PMC12667530; doi:10.1002/advs.202510338)
Supplement: Supplementary file 1 — Supporting Information [file ADVS-12-e10338-s002.pdf]

Supplementary Information:

Ultrafast Dynamic Defect Inspection with Computational Neuromorphic Imaging

Shuo Zhu[1,*], Qianfeng Yin[2,3], Chutian Wang[1], Jianqing Huang[4], and Edmund Y. Lam[1,*]

[1]Department of Electrical and Electronic Engineering, The University of Hong Kong, Pokfulam, Hong Kong SAR, China
[2]Meta-force Institute of Computation and Information (MICI), Hong Kong SAR, China.
[3]Hong Kong Industrial Artificial Intelligence and Robotics Centre (FLAIR), Hong Kong SAR, China.
[4]School of Aerospace Engineering, Xiamen University, Xiang'an South Road, Xiang'an District, Xiamen 361005, Fujian, China.
[*]e-mail: zhushuo@hku.hk; elam@eee.hku.hk

Contents

Supplementary Note 1: Experimental set-up

Supplementary Note 2: Event generation and compensation

Supplementary Note 3: Defect image formulation

Supplementary Note 4: Additional results under challenging illumination conditions

Supplementary Note 5: Defect edge representation with events

Supplementary Note 6: Events representation with vibration

Supplementary Note 7: Inspection resolution

Supplementary Note 8: Comparison of existing enhancement method

Supplementary Note 9: Additional results of practical samples

Supplementary Note 1: Experimental set-up

The photograph of the experimental set-up and details are provided in Supplementary Fig. 1, which employs a bright field illumination scheme like Supplementary Fig. 2a. We constructed a prototype for laboratory validation, demonstrating that the CNI-informed method outperforms traditional methods in various complex environments. An LED is used to illuminate the inspected samples with the coaxial forward lighting approach and can be adjusted to control the lighting luminance. For higher magnification objectives, i.e., 20X objective lens, the resolution is higher, the corresponding field of view is smaller, and the working distance is reduced to 10.4 mm. Here the translation stage is WN262TA20, the working stroke is 20 mm, the maximum speed is 10 mm/s and the repeatability is 2 µm. To be more specific, the cost of the event camera used in this manuscript is about $4500, which can support simultaneous frame and event output. It is convenient for concept verification and experimental comparison quickly with these two different modes. The processing of events is currently done only through the CPU, and this work was carried out on a laptop (Intel Core i7-13700H) with no special requirements for computing resources.

To emphasize the advantages of event cameras over conventional and high-speed cameras, we present the following parameters. As shown in Supplementary Table 1, we use event sensors to capture more efficient dynamic information than conventional and high-speed cameras. The event cameras have a higher temporal resolution than conventional frame-based cameras.

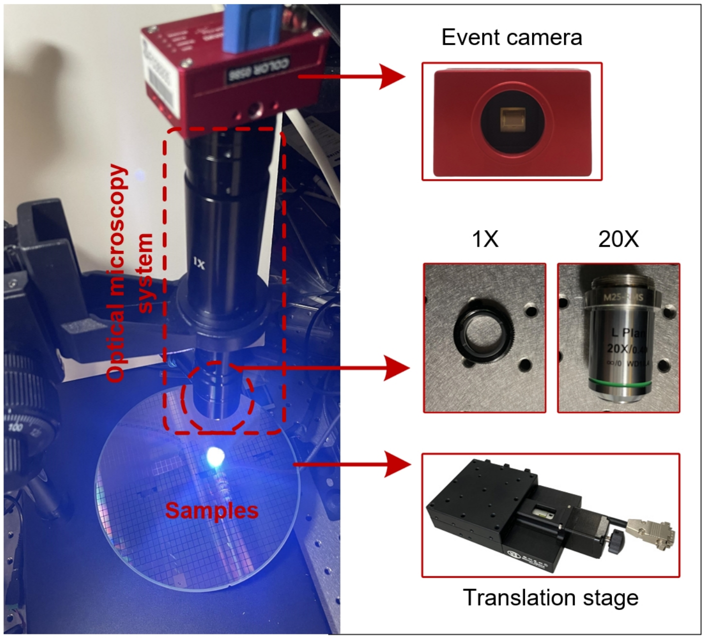

Supplementary Fig. 1. Experimental set-up. The imaging system consists of an incoherent light source, a lens, and a camera. The transmissive object on a negative resolution test target is hidden in the scattering sample and is moved via a two-axis motorized precision translation stage.

The CNI-informed inspection method using an event camera can be applied to various illumination modes in

Supplementary Table. 1. Conventional frame-based camera *vs* Event cameras

**parameters**[1]	**Frame**	**Event**
fps/measurement rate	$\leqslant$ 1000 fps	$>$ 10 000 fps
dynamic range	60 dB to 100 dB	110 dB to 140 dB
data rate[2]	10 MB/s to 40 MB/s	1 MB/s to 10 MB/s
power consumption	1 W to 10 W	0.01 mW to 10 mW
weight	200 g to 700 g	80 g to 110 g

[1] Specific values may vary with models and types.
[2] It depends on dynamics and scenes for event cameras.

inspection scenarios. As shown in Supplementary Fig. 2, four different illumination approaches are presented for the feasible inspection modes with the CNI techniques. The event camera's high temporal resolution, high dynamic range, and other advantages enable it to work well between different illumination modes and provide better robustness to different complex samples. For example, under dark-field illumination like Supplementary Fig. 2b, the event camera has a more sensitive logarithmic response in dark lighting conditions, thereby enabling a more accurate diagnosis of defects through their motion patterns in that scene.

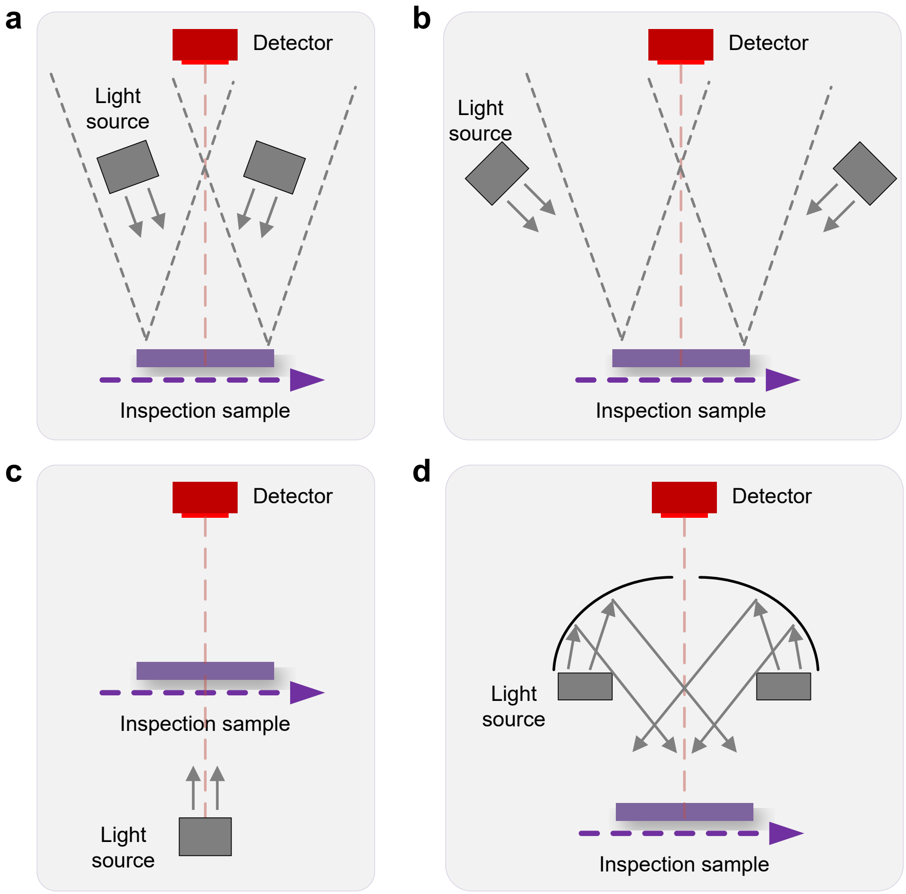

Supplementary Fig. 2. Schematic diagram of typical illumination modes. (a) Bright-field illumination. (b) Dark-field illumination. (c) Backlight illumination. (d) Diffuse illumination.

Supplementary Note 2: Event generation and compensation

Event cameras have independent pixels that respond asynchronously and trigger an event $e_k = (\mathbf{x}_k, p_k, t_k)$ is triggered as soon as the logarithmic brightness change $L(\mathbf{x}_k, t_k)$ exceeds a preset threshold C at the timestamp t_k

$$\Delta L(\mathbf{x}_k, t_k) \triangleq L(\mathbf{x}_k, t_k) - L(\mathbf{x}_k, t_k - \Delta t_k) = p_k C \tag{1}$$

where $\mathbf{x}_k = (x_k, y_k)$ is the spatio-temporal coordinates, and $\Delta L(\mathbf{x}_k, t_k)$ represents the recorded temporal increment with the time elapsed Δt_k since the last event at the same pixel $\mathbf{x}_k$[1]. The event stream data encodes the brightness changes' time, location, and polarity. Motion compensation approaches have been recently introduced for processing the visual information acquired by event cameras, which is a crucial step for downstream tasks[2]. Events are warped along motion trajectories whose parameters depend on the estimated quantity.

Here, we leverage the contrast maximization (Cmax) framework for motion composition[3]. The conventional contrast maximization applies an affine transformation $\mathscr{A}$ to the event clusters $\mathscr{E}$ to produce a geometrically warped event cluster $\mathscr{E}' \triangleq \{e'_k\}_{k=1}^{N_e}$, with each point representing the warped event e'_k. The warping transformation can be defined as[4]

$$\mathbf{x}'_k(\mathbf{v}) = \mathscr{A}(\mathbf{v}; \mathbf{x}_k, t_k) = \mathbf{x}_k - (t_k - t_{ref})\mathbf{v} \tag{2}$$

where the flow $\mathbf{v}$ serves as the warping parameter for defect reconstruction.

Aggregating all the events in $\mathscr{E}'$ yields an accumulated sample image, namely an image of warped events (IWE), i.e.,

$$\Delta \hat{L}(\mathbf{x}; \mathbf{v}) = \int_{t_{ref}}^{t_{ref}+\Delta t} e'_k(\mathbf{x}'_k(\mathbf{v}), p_k, t_k) \mathrm{d}t \tag{3}$$

where $e'_k(\mathbf{x}'_k(\mathbf{v}), p_k, t_k) = C p_k \delta\left(\mathbf{x} - \mathbf{x}'_k(\mathbf{v})\right) \delta(t - t_k)$ represents the discrete warped event by applying the affine transformation $\mathscr{A}$, with $\delta(\cdot)$ being the Kronecker delta function.

The estimated optimal flow can be solved by maximizing the sharpness of the image of warped events (IWE), which is characterized as the variance

$$\mathbf{v}^* = \max_{\mathbf{v}} \frac{1}{|\Omega|} \int_{\Omega} \left(\Delta \hat{L}(\mathbf{x}; \mathbf{v}) - \mu\left(\Delta \hat{L}(\mathbf{x}; \mathbf{v})\right) \right)^2 \mathrm{d}\mathbf{x} \tag{4}$$

where $|\Omega|$ is the area within the lenslet boundary, $\mu(\cdot)$ represents the statistical mean.

Then we can obtain the optimized signals for the next defect inspection and analysis with the warped events. The warped events have sharp visualization and represent the strength of the moving edges in the testing samples, which is helpful for further defect inspection and analysis.

Supplementary Note 3: Defect image formulation

The CNI-informed defect inspection is a new topic with challenges because event-stream data is an unfamiliar representation of visual datasets, which depend on motion and exhibit a considerable amount of noise and non-ideal effects. Therefore, we can analyze the defect with the conventional approach by formulating event-based images.

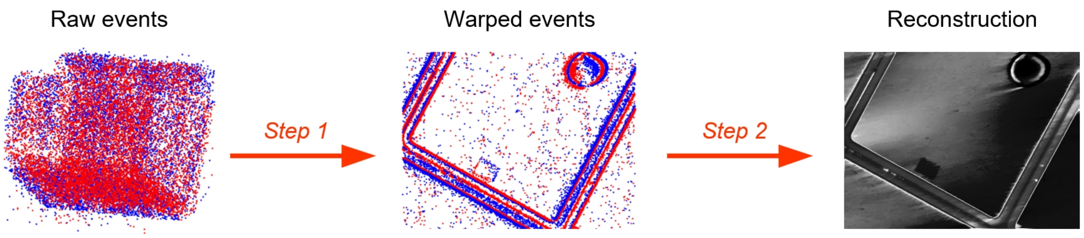

Supplementary Fig. 3. Defect image formulation from events to brightness. Step 1 is using Cmax to warp the raw events and obtain the motion information. Step 2 is the image reconstruction process with regularization using optical flow.

We first tackle the combined problem of optical flow estimation and motion compensation, which leads us to formulate event-based image reconstruction as a linear inverse problem with deep regularization[5]. As the clearest IWE estimates the motion-corrected increment within Δt, its spatial distribution can be physically interpreted as the gradient according to the known optical-flow constraint[5].

$$\Delta\hat{L}(\mathbf{x};\mathbf{v}^*) \approx \Delta L(\mathbf{x}) = -\nabla L(\mathbf{x})\cdot\mathbf{v}^*\Delta t \tag{5}$$

where $\nabla L(\mathbf{x})$ represents the gradient of the logarithmic intensity.

As such, the optimal logarithmic intensity of the defect image can be solved by minimizing the data fidelity cost function

$$L^*(\mathbf{x}) = \arg\min_{L}\frac{1}{2}||\Delta\hat{L}(\mathbf{x};\mathbf{v}^*)+\nabla L(\mathbf{x})\cdot\mathbf{v}^*\Delta t||_2^2+\kappa_1||\nabla L(\mathbf{x})||_1+\kappa_2||\nabla^2 L(\mathbf{x})||_1 \tag{6}$$

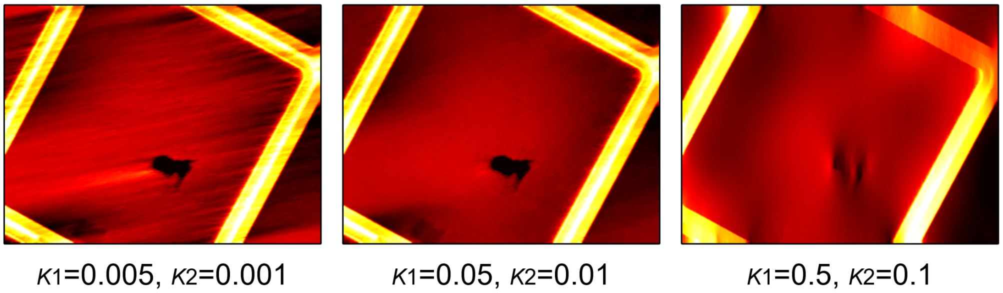

Supplementary Fig. 4. Effect of varying the regularizer weight for defect images reconstruction. The motion speed of this sample is 7 mm/s.

where the first term is the data fidelity term, $||\cdot||_p$ represents the p-norm, and κ_1, κ_2 are the weights of the regularizers to control the degree of regularization on the defect image. As shown in Supplementary Fig. 3, the defect image is reconstructed with two regularization terms.

Here, the main hyperparameters of the method are the event number and regularizer weights $\kappa_1 = 0.05$, $\kappa_2 = 0.01$. The event numbers mainly depend on the amount of texture in different scenes (e.g., the motion speed and illumination conditions), which has been discussed with time windows in the manuscript. As shown in Fig 4, the impact of varying regularizer weights highlights a trade-off between artifact removal, detail preservation, and over-smoothing. A small weight (left) preserves details but fails to eliminate artifacts in flow streamlines. A large weight (right) leads to over-smoothing, capturing only prominent edges. An intermediate weight (middle) strikes a balance, optimizing both artifact removal and detail preservation. Further optimization information, analysis, and released code can be referred to formulating event-based image reconstruction as a linear inverse problem[5].

Supplementary Note 4: Additional results under challenging illumination conditions

As shown in Supplementary Fig 5, we add additional experimental results and analysis of various defect types to prove it more comprehensively. The light scratches and contaminant defects are inspected with the CNI-informed method under different illumination conditions, which can obtain reliable visual performance and robust defect information than the frame-based approach. Furthermore, as shown in Supplementary Fig 6, we implemented defect visualization under lower illumination conditions (i.e., 0.097 lux). Lower illumination conditions could make it more difficult to generate events, which affects the visualization of defects. However, improving the imaging quality to a certain extent is possible by accumulating more events by increasing the value of the time window.

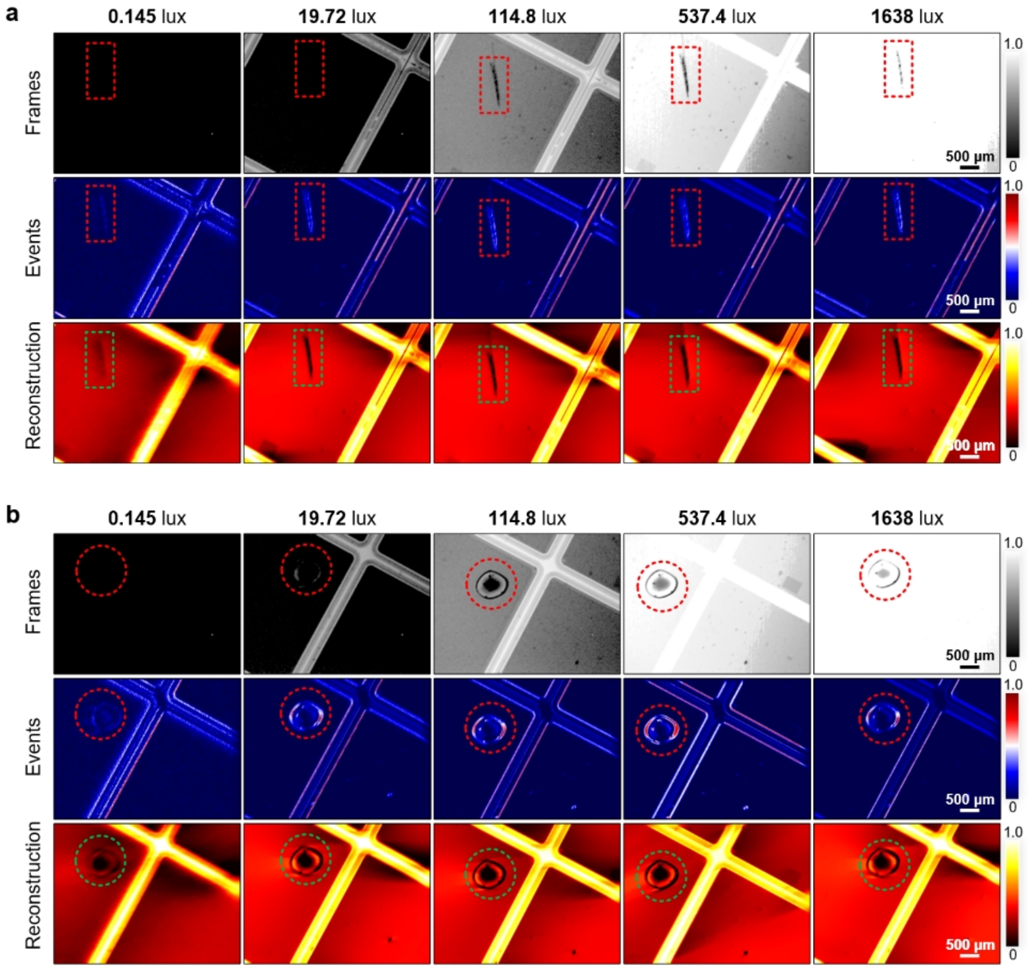

Supplementary Fig. 5. More examples are implemented for the challenging dynamic range inspection. a) and b) are light scratches and contaminant defects under different illumination conditions.

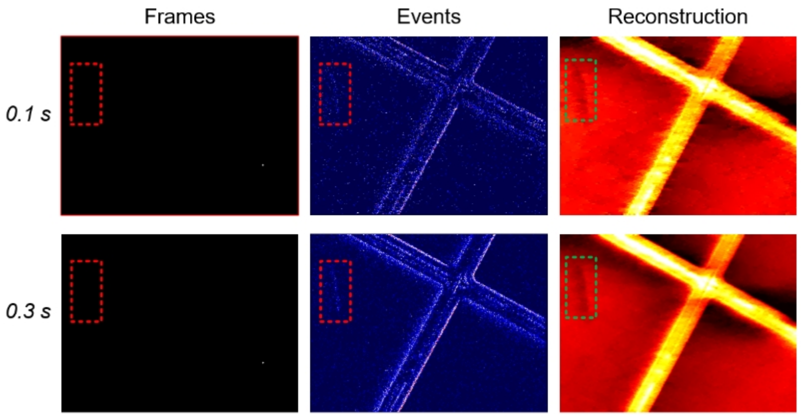

Supplementary Fig. 6. Defect visualization under lower illumination condition (i.e., 0.097 lux). The first and second lines are to select the window to 0.1 and 0.3 s.

Supplementary Note 5: Defect edge representation with events

Events along accurately estimated motion trajectories are distinct and align precisely with edges. Events are primarily triggered by the motion of the sample's edges. Thus, these events can be efficiently used to characterize the edge information of the sample. When the sample is stationary or moving slowly, conventional frame-based methods can obtain relatively reliable edges of the sample. However, when the sample moves faster, frame-based edge detection methods struggle to obtain effective edge information. In contrast, the event-based method can still directly obtain reliable edge results from fast-moving samples, capturing high-frequency information.

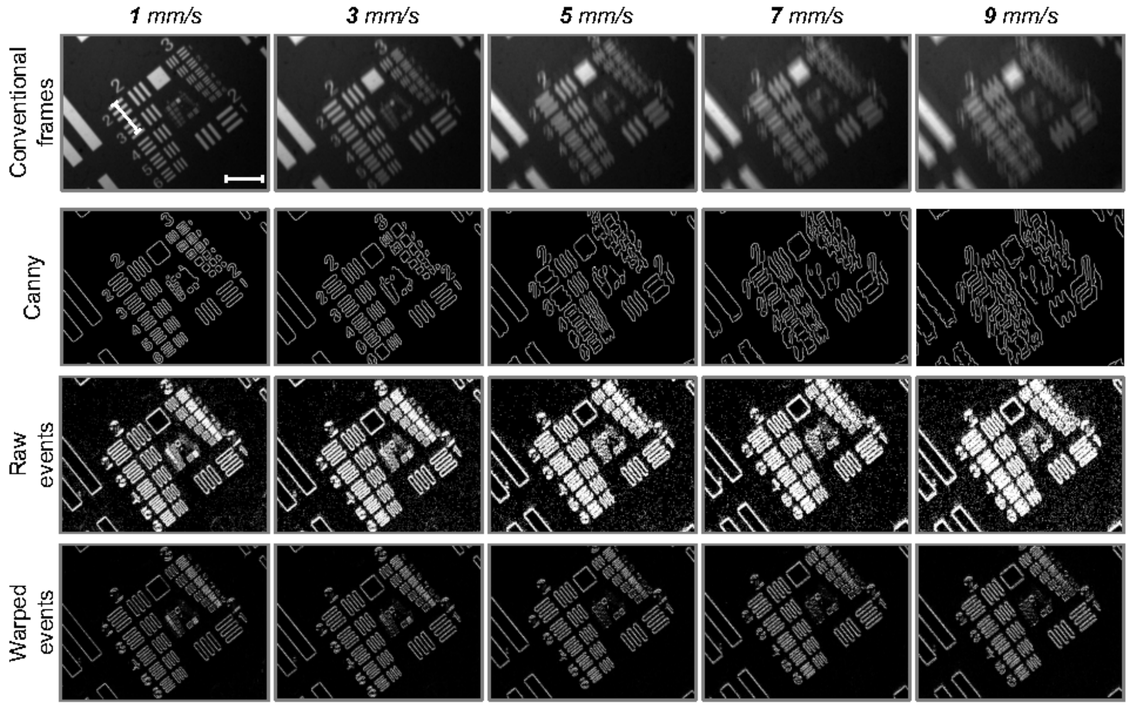

Supplementary Fig. 7. Warped events captured by the event camera can be used directly to characterize the edge information of the samples.

As shown in Supplementary Fig. 7, the raw data captured by the event camera can be directly used to describe the edge information of the sample. The high temporal resolution of The event camera allows for more accurate and reliable characterization of the edges of moving samples. As shown in Supplementary Fig. 8, the experimental results under different lighting conditions are similar to the above, and the proposed method can help to obtain the defect edge information in highly dynamic scenes. We have analyzed and compared both ultrafast speed motion samples and high dynamic range samples under various lighting conditions. Our method is robust enough to directly visualize the edge information of the samples in both high-speed motion and high dynamic range scenarios, across various lighting conditions, significantly improving the efficiency of sample inspection and diagnostics.

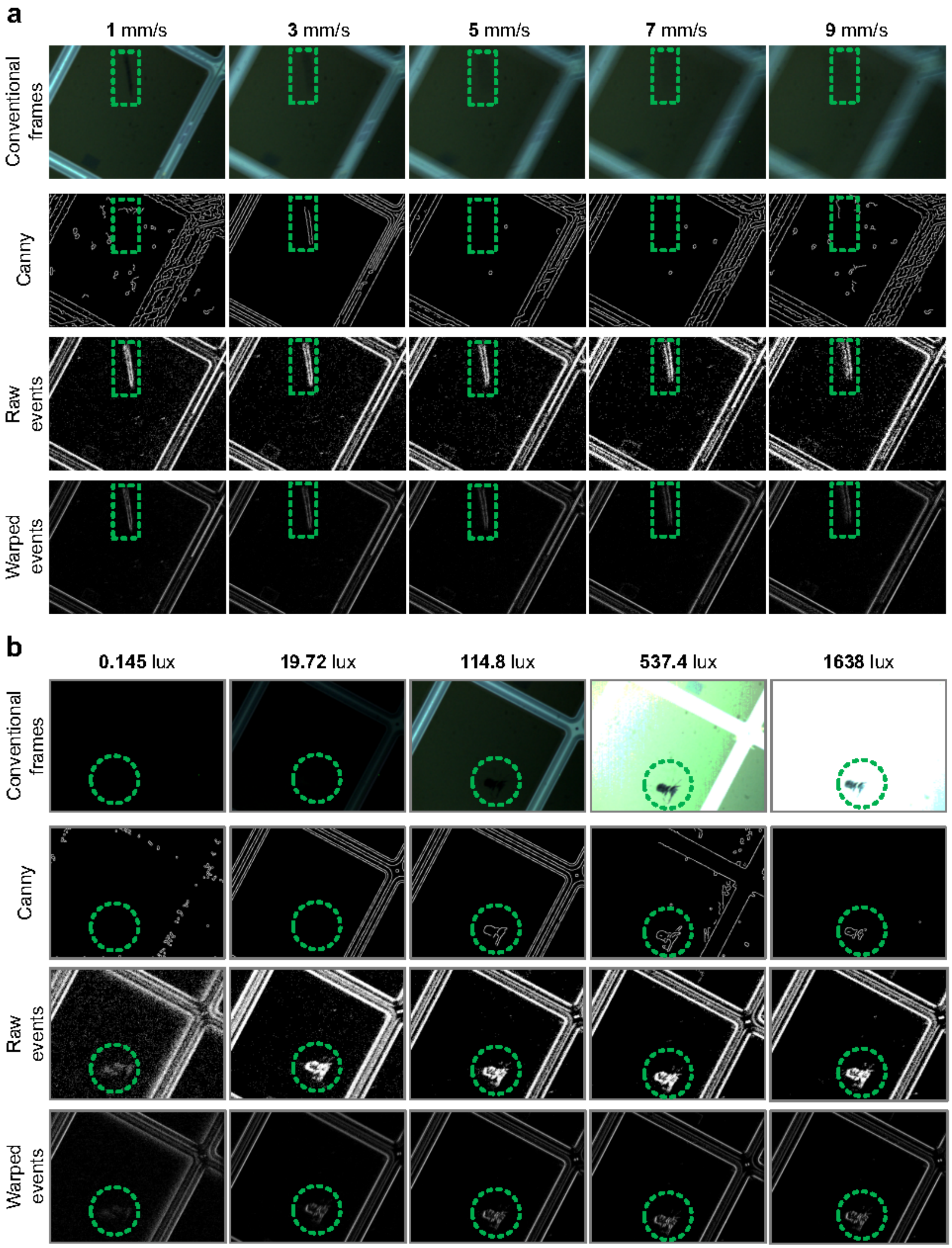

Supplementary Fig. 8. Direct edge visualization of the practical samples in high-speed motion and various illumination conditions.

Supplementary Note 6: Event representation with vibration

Experimental results are presented to analyze the resolution of our inspection method. We propose using a vibration mechanism to generate event records, which are then processed to reconstruct a typical defect image. The relative motion from vibration creates events that, when processed using the CNI technique, contain sample structure information for high-magnification chip defect diagnosis. By utilizing these events, we enhance our characterization of the events and reconstruct the structural details relevant to sample defects. The reconstruction findings demonstrate that the structural information can be effectively restored, attributed to the high temporal resolution of the event camera. Leveraging vibration-induced events for image reconstruction and defect detection provides enhanced visualization and resolution compared to conventional direct acquisition methods.

To compare imaging results across vibration frequencies, we fixed the displacement stage and adjusted motor speeds to generate varying frequencies. Consistent with prior experiments, we reduced time windows for high-frequency states proportionally as speed increased, with rates inversely proportional to speed magnitude. As shown in Supplementary Fig. 9, CNI-informed zoomed-in regions exhibit enhanced visual clarity and superior high-contrast resolution. These visualization outcomes confirm that the CNI-informed approach surpasses traditional frame-based techniques in resolution capabilities. A stabilization strategy for processing events is needed to effectively leverage vibration, whether natural or induced, for defect inspection.

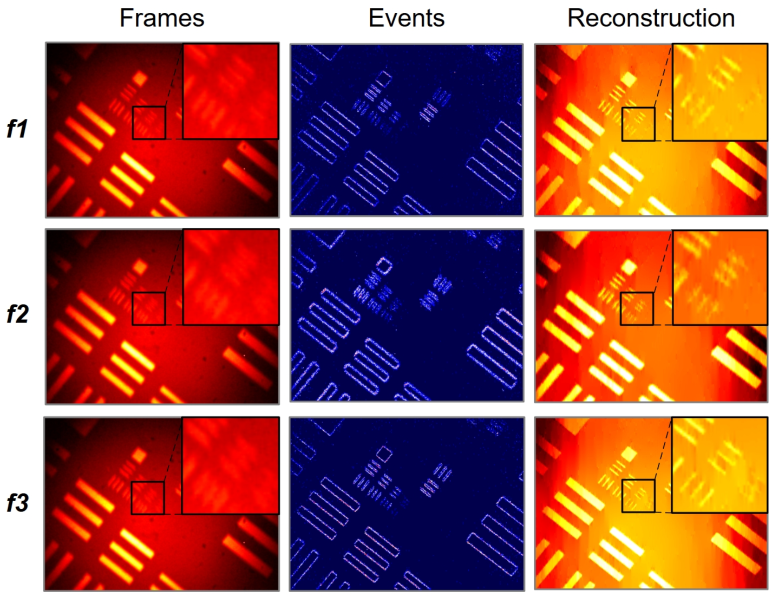

Supplementary Fig. 9. Defect image formulation from events with different vibration frequencies. Three different frequencies are controlled with corresponding motion settings, i.e., motor speeds with 0.5 mm/s, 1 mm/s, and 2 mm/s.

Supplementary Note 7: Inspection resolution

Motion blur can significantly impact defect diagnosis resolution in some scenarios requiring fast motion detection. The resolution board was utilized to compare image and event detection results at varying motion speeds and illumination conditions. As shown in Supplementary Fig. 10, in high-speed, high-dynamic-range scenarios, the image resolution of the inspection is effectively guaranteed. For example, at a speed of 5 mm/s, the image resolution is approximately 3 times than conventional frames. We evaluate the motion resolution under different lighting conditions and observe the evident detection advantage over the traditional method. In contrast, the method presented in this paper exhibits improved resolution and robustness against high-speed motion. This method is more suitable for the rapid dynamic monitoring capability in real-world scenarios.

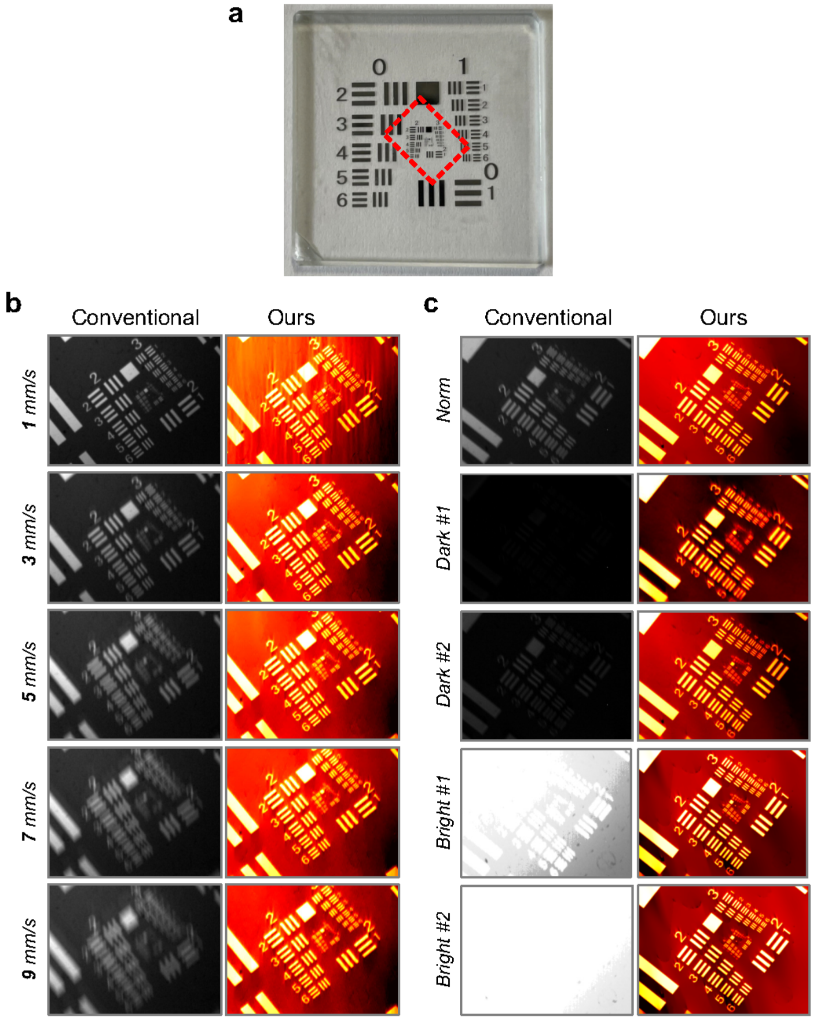

Supplementary Fig. 10. Detection results of the resolution version at different motion speeds.

Supplementary Note 8: Comparison of existing enhancement method

Post-processed frames using deblurring algorithms can enhance defect visualization in moving samples. Existing computational deblurring methods, particularly those employing blind convolution, have shown some success in mitigating motion blur. For comparison, we apply a blind image deblurring method with a local maximum gradient prior[6] to evaluate its performance against our proposed method. The results, illustrated in Supplementary Fig 10, demonstrate that our method achieves more stable and reliable target reconstruction across various motion scenarios, with superior image resolution. Additionally, comparative results highlight the efficiency and accuracy of our approach in inspecting fast-moving samples.

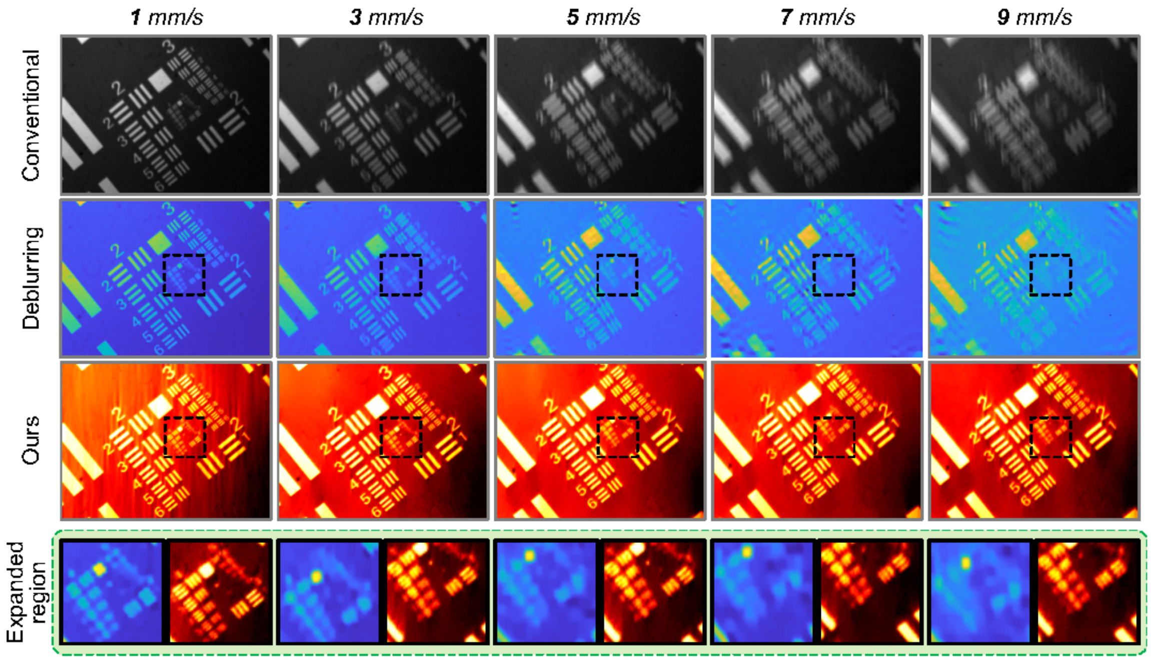

Supplementary Fig. 11. Comparison results of the deblurring method and ours at different motion speeds.

Supplementary Note 9: Additional results of practical samples

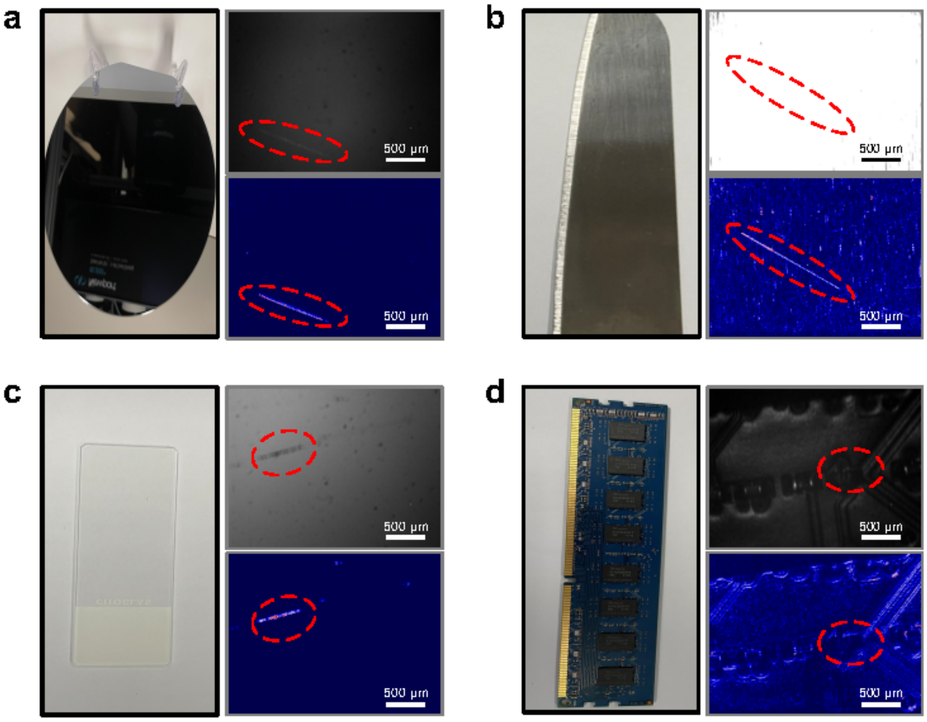

Supplementary Fig. 12. Detection results for different samples with the same device parameter settings. (a) to (d) Wafers, metal tools, glass slides, and memory stick PCBs at a time. Detection results were obtained for various samples using identical device parameter settings, including wafers, metal tools, glass slides, and memory stick PCBs.

The method proposed in this paper has better robustness properties across diverse samples, enabling accurate defect inspection without requiring adjustments to the optical device and sensors. The inspection accuracy and results will not be affected by factors such as the target's different reflective materials, which are suitable for more complex multi-category target defect inspection. As shown in Supplementary Fig 12, the method introduced in this study exhibits enhanced robustness across various samples, enabling accurate detection without changing the detection device. Inspection accuracy and results remain unaffected by factors like diverse refractive materials present in the target, making it well-suited for complex multi-category target defect detection.

Further, a more complex sample is also verified with the CNI-informed inspection method. As shown in Supplementary Fig 13, we evaluated our CNI approaches in a circuit with multiple target components. Our method can obtain robust capability with different reflective characteristics of components. This comparison underscores CNI's robustness in dynamic environments and complex samples. We inspect the complex circuit with an industrial lens (ZLKC, VM12120MPC) to verify the robustness and extension capability. The sample with multiple target components is inspected with a handheld scan, then the target information is collected be recorded quickly via our CNI approach. Therefore, the CNI-formed technique offers flexible and extendable

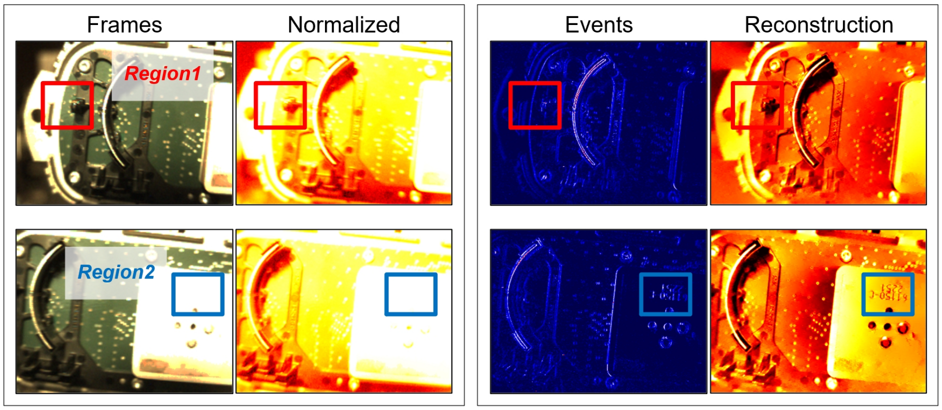

Supplementary Fig. 13. Experimental results in a complex circuit with multiple target components.

capabilities for various vision/imaging-based inspection systems.

References

1. Gallego, G., Forster, C., Mueggler, E. & Scaramuzza, D. Event-based camera pose tracking using a generative event model. *arXiv preprint arXiv:1510.01972* (2015).
2. Gallego, G., Gehrig, M. & Scaramuzza, D. Focus is all you need: Loss functions for event-based vision. In *Proceedings of the IEEE/CVF Conference on Computer Vision and Pattern Recognition*, 12280–12289 (2019).
3. Gallego, G., Rebecq, H. & Scaramuzza, D. A unifying contrast maximization framework for event cameras, with applications to motion, depth, and optical flow estimation. In *Proceedings of the IEEE Conference on Computer Vision and Pattern Recognition*, 3867–3876 (2018).
4. Gehrig, D., Rebecq, H., Gallego, G. & Scaramuzza, D. Eklt: Asynchronous photometric feature tracking using events and frames. *Int. J. Comput. Vis.* **128**, 601–618 (2020).
5. Zhang, Z., Yezzi, A. J. & Gallego, G. Formulating event-based image reconstruction as a linear inverse problem with deep regularization using optical flow. *IEEE Transactions on Pattern Analysis Mach. Intell.* **45**, 8372–8389 (2022).
6. Chen, L., Fang, F., Wang, T. & Zhang, G. Blind image deblurring with local maximum gradient prior. In *Proceedings of the IEEE/CVF conference on computer vision and pattern recognition*, 1742–1750 (2019).
